# GA^2^LEN ANACARE consensus statement: Potential of omalizumab in food allergy management

**DOI:** 10.1002/clt2.70002

**Published:** 2024-11-06

**Authors:** Torsten Zuberbier, Antonella Muraro, Ulugbek Nurmatov, Stefania Arasi, Katarina Stevanovic, Aikaterini Anagnostou, Roberta Bonaguro, Sharon Chinthrajah, Gideon Lack, Alessandro Fiocchi, Thuy‐My Le, Paul Turner, Montserrat Alvaro Lozano, Elizabeth Angier, Simona Barni, Phillippe Bégin, Barbara Ballmer‐Weber, Victoria Cardona, Carsten Bindslev‐Jensen, Antonella Cianferoni, Nicolette de Jong, Debra de Silva, Antoine Deschildre, Audrey Dunn Galvin, Motohiro Ebisawa, David M. Fleischer, Jennifer Gerdts, Mattia Giovannini, Josefine Gradman, Susanne Halken, Syed Hasan Arshad, Ekaterina Khaleva, Susanne Lau, Richard Loh, Mika J. Mäkelä, Mary Jane Marchisotto, Laura Morandini, Charlotte G. Mortz, Caroline Nilsson, Anna Nowak‐Wegrzyn, Marcia Podestà, Lars K. Poulsen, Graham Roberts, Pablo Rodríguez del Río, Hugh A. Sampson, Angel Sánchez, Sabine Schnadt, Peter K. Smith, Hania Szajewska, Natasa Teovska Mitrevska, Alice Toniolo, Carina Venter, Amena Warner, Gary W. K. Wong, Robert Wood, Margitta Worm

**Affiliations:** ^1^ Institute of Allergology Charité ‐ Universitätsmedizin Berlin Berlin Germany; ^2^ Fraunhofer Institute for Translational Medicine and Pharmacology ITMP Immunology and Allergology Berlin Germany; ^3^ Food Allergy Referral Centre Department of Women and Children Health Padua University Hospital Padua Italy; ^4^ Division of Population Medicine School of Medicine Cardiff University Cardiff UK; ^5^ Allergy Unit Pediatric Hospital Bambino Gesù IRCCS Rome Italy; ^6^ Department of Pediatrics Division of Allergy and Immunology Baylor College of Medicine Houston Texas USA; ^7^ Sean N. Parker Center for Allergy and Asthma Research at Stanford University Stanford California USA; ^8^ Paediatric Allergy King's College London London UK; ^9^ Department of Women and Children's Health/Peter Gorer Department of Immunobiology School of Life Course and Population Sciences King's College London London UK; ^10^ Evelina London Children's Hospital Guy's and St. Thomas' NHS Foundation Trust London UK; ^11^ Department of Dermatology/Allergology University Medical Center Utrecht Utrecht University Utrecht Netherlands; ^12^ Centre of Translational Immunology University Medical Center Utrecht Utrecht University Utrecht Netherlands; ^13^ Imperial College London London UK; ^14^ Pediatric Allergy and Clinical Immunology Department Hospital Sant Joan de Déu Barcelona Spain; ^15^ Institut de Recerca Sant Joan de Déu Barcelona Spain; ^16^ Universitat de Barcelona Barcelona Spain; ^17^ Primary Care Population Science and Medical Education Faculty of Medicine University of Southampton Southampton UK; ^18^ Allergy Unit Meyer Children's Hospital IRCCS Florence Italy; ^19^ Section of Allergy and Clinical Immunology Department of Pediatrics Centre Hospitalier Universitaire Sainte‐Justine Montreal Quebec Canada; ^20^ Department of Medicine Section of Allergy and Clinical Immunology Centre Hospitalier de l'Université de Montréal Montreal Quebec Canada; ^21^ Clinic for Dermatology and Allergology St. Gallen Switzerland; ^22^ Department of Dermatology University Hospital Zurich Zurich Switzerland; ^23^ Allergy Section Hospital Universitari Vall d'Hebron Barcelona Spain; ^24^ Department of Dermatology and Allergy Centre Odense Research Centre for Anaphylaxis (ORCA) Odense University Hospital University of Southern Denmark Odense Denmark; ^25^ Allergy and Immunology Division Perelman School of Medicine The Children's Hospital of Philadelphia University of Pennsylvania Philadelphia Pennsylvania USA; ^26^ Internal Medicine Allergology & Clinical Immunology Erasmus MC University Medical Center Rotterdam Rotterdam The Netherlands; ^27^ The Evidence Centre London UK; ^28^ University Lille INSERM Unit 1019 CNRS UMR 9017 CHU Lille Institut Pasteur de Lille Center for Infection and Immunity of Lille Lille France; ^29^ Allergy Research Group University College Cork Cork Ireland; ^30^ Clinical Research Center for Allergy and Rheumatology NHO Sagamihara National Hospital Kanagawa Japan; ^31^ University of Colorado Denver School of Medicine Children's Hospital Colorado Aurora Colorado USA; ^32^ Food Allergy Canada Toronto Ontario Canada; ^33^ Hans Christian Andersen Children's Hospital Odense University Hospital Odense Denmark; ^34^ Clinical and Experimental Sciences Faculty of Medicine University of Southampton Southampton UK; ^35^ David Hide Asthma and Allergy Research Centre Isle of Wight UK; ^36^ NIHR Southampton Biomedical Research Centre Southampton General Hospital Southampton UK; ^37^ Faculty of Medicine University of Southampton Southampton UK; ^38^ Department of Pediatric Respiratory Medicine, Immunology and Critical Care Medicine Charité Universitätsmedizin Berlin Berlin Germany; ^39^ Medical School The University of Western Australia Perth Western Australia Australia; ^40^ Perth Children's Hospital Perth Western Australia Australia; ^41^ Australasian Society of Clinical Immunology and Allergy (ASCIA) Sydney Western Australia Australia; ^42^ Skin and Allergy Hospital Helsinki University Central Hospital and University of Helsinki Helsinki Finland; ^43^ MJM Advisory New York New York USA; ^44^ Department of Clinical Science and Education Karolinska Institutet Stockholm Sweden; ^45^ Sachs Children and Youth Hospital South Hospital Stockholm Sweden; ^46^ Department of Pediatrics Hassenfeld Children's Hospital NYU Grossman School of Medicine New York City New York USA; ^47^ Department of Pediatrics Gastroenterology and Nutrition Collegium Medicum University of Warmia and Mazury Olsztyn Poland; ^48^ EFA ‐ European Federation of Allergy and Airways Diseases Patients' Associations Brussels Belgium; ^49^ Allergy Clinic Copenhagen University Hospital at Herlev‐Gentofte Copenhagen Denmark; ^50^ Department of Paediatric Allergy and Respiratory Medicine University of Southampton Southampton UK; ^51^ NIHR Southampton Biomedical Research Centre Southampton UK; ^52^ David Hide Asthma and Allergy Centre St Mary Hospital Isle of Wight UK; ^53^ Hospital Infantil Universitario Niño Jesús Madrid Spain; ^54^ Division of Allergy and Immunology Department of Pediatrics Jaffe Food Allergy Institute Icahn School of Medicine at Mount Sinai New York New York USA; ^55^ AEPNAA Spanish Association of People with Food and Latex Allergy Madrid Spain; ^56^ German Allergy and Asthma Association (DAAB) Mönchengladbach Germany; ^57^ Griffith University School of Medicine Gold Coast Queensland Australia; ^58^ Department of Paediatrics Medical University of Warsaw Warszawa Poland; ^59^ Dermatology Department Remedika General Hospital Skopje North Macedonia; ^60^ Department of Dermatology International Balkan University Skopje North Macedonia; ^61^ Allergy UK London UK; ^62^ Chinese University of Hong Kong Hong Kong Hong Kong; ^63^ Department of Pediatrics John Hopkins University School of Medicine Baltimore Maryland USA; ^64^ Division of Allergy and Immunology Department of Dermatology, Allergy and Venerology Charité Universitätsmedizin Berlin Berlin Germany

**Keywords:** anaphylaxis, desensitization, food allergy, immunoglobulin E, omalizumab

## Abstract

Immunoglobulin E (IgE)‐mediated food allergies are the most common type of food allergy, often causing rapid symptoms after exposure to allergens posing a serious health risk and a high impact on patient's and caregiver's quality of life. Omalizumab, a humanized anti‐IgE monoclonal antibody, reduces allergic reactions by binding to circulating IgE. Omalizumab has been successfully used in allergic asthma, chronic rhinosinusitis with nasal polyps, and chronic urticaria, and was recently approved for treating IgE‐mediated food allergies by the US Food and Drug Administration (FDA). This GA^2^LEN ANACARE Consensus Statement presents our position on the use of omalizumab for treating IgE‐mediated food allergies, based on a systematic review and meta‐analysis, experience with use for other conditions, and expert consensus achieved via an eDelphi process. Following publication of the recent OUtMATCH study (stage 1) results and subsequent FDA approval, we propose that there is now sufficient evidence to recommend omalizumab as the only drug currently available that can mechanistically reduce IgE‐mediated food allergic reactions. We acknowledge that the evidence does not reach the highest level of evidence which would be needed for a guideline recommendation.

## 1 INTRODUCTION

GA^2^LEN, the Global Allergy and Asthma Excellence Network, was created in 2004 as the European Union network of excellence in collaboration with EAACI (European Academy of Allergy and Clinical Immunology). Currently, it is the largest and most active worldwide multidisciplinary network of clinical and research centers in allergy and asthma. An important GA^2^LEN activity is knowledge‐exchange to progress novel and emerging approaches to treat severely affected patients. Here, the GA^2^LEN ANACARE centers of reference and excellence for anaphylaxis and food allergy outline our consensus statement on the use of omalizumab for treating IgE‐mediated food allergy, acknowledging its existing licenses for other allergic diseases and its recent approval for IgE‐mediated food allergy by the US Food & Drug Administration (FDA). We summarize our current position on the use of this biologics, and outline current evidence gaps which preclude a more comprehensive set recommendations.

## 2 CHALLENGES OF FOOD ALLERGY

The number of people diagnosed with an immediate‐type IgE‐mediated food allergy is increasing. The prevalence and types of food allergies vary globally, but up to 6% of children and 3% of adults may be affected by allergies to cow's milk, hen's egg, peanuts, sesame, wheat, and other foods, which has consequences on those providing safe food options for them.^1^ While most reactions are not life‐threatening, severe anaphylaxis reactions do occur, which can be fatal. Our current inability to identify those at greatest risk of more severe reactions means that people with food allergy are often managed as being at equal risk of fatal reactions, causing unnecessary anxiety and excessive dietary restriction.

Until recently, the mainstay of management was to avoid the trigger food allergens and prescribe adrenaline (epinephrine) autoinjectors for use in the event of an allergic reaction. However, this is not always easy in day‐to‐day life. It can be difficult to avoid “hidden” foods and there is always a danger of cross contamination. Adults and children with food allergies can become fearful of having a reaction, which can affect their nutrition, health, and mental wellbeing.^2^ The aim of GA^2^LEN is to investigate and offer the best treatment options available. In our understanding, simple avoidance measures are not in line with the World Health Organization (WHO) standards, which state that everyone deserves the highest level of health.

Food allergies have been shown to significantly affect the quality of life in patients of all ages, and the mental wellbeing of the whole family.^3^, ^4^, ^5^ Management involving avoidance of allergen(s) by eating a very restricted diet can adversely impact health and nutrition such as creating gut inflammation and barrier dysfunction.^6^, ^7^ Fear of a potential reaction when in social situations, particularly those that involve food, may lead individuals and their families to greatly restrict their, or their child's social lives.^6^, ^8^

By definition, IgE‐mediated allergy requires the presence of a specific antigen as well as a specific IgE antibody recognizing this antigen. Therefore, reducing serum IgE offers another approach (alongside allergen avoidance) to reduce the risk of an acute allergic reaction. In this position paper, we assess evidence relating to the use of omalizumab for specific patient subgroups that have a difficulty avoiding their food allergen, for example, people with multiple food allergies, those who have a very low reaction threshold and may therefore be prone to react to low‐level allergen contamination, as well as higher risk patients such as those living in remote areas without access to emergency care. We also explore the use of omalizumab as an adjunct therapy in patients who fail to achieve successful desensitization with food allergy immunotherapy due to frequent adverse events.

## 3 BACKGROUND INFORMATION ON OMALIZUMAB

In IgE‐mediated food allergy, mediators such as histamine and leukotrienes are released from mast cells, which in turn cause clinical symptoms. Omalizumab (Xolair) is a commercially available humanized anti‐IgE monoclonal antibody that interferes with the body's immune system to reduce people's sensitivity to allergens. It was first approved by the FDA for moderate‐severe asthma in 2003; subsequently, the approved indications have broadened and now include childhood asthma, chronic rhinosinusitis with nasal polyps, chronic urticaria and – since February 2024 – for IgE‐mediated food allergy in certain adults and children aged one year or older.^9^

Due to its anti‐IgE mechanism of action, it helps to reduce immune responses in people with IgE‐mediated food allergies. Omalizumab binds to circulating IgE, reducing IgE receptor expression and decreasing mediator release from mast cells and basophils. In food allergy, omalizumab may also exert its protective effect through the formation of allergen‐specific circulating IgE‐IgG complexes, which compete with cell‐bound IgE for epitopes on allergen surface, thus disrupting IgE receptor aggregation.^10^, ^11^, ^12^ Since omalizumab is a nonspecific anti‐IgE antibody and inhibiting IgE is the basic principle of treating a type I allergic reaction, this medicine is not restricted to specific allergens and may be effective for people with multiple food allergies, commonly found in clinical practice. The principle of IgE‐mediated allergic reactions is conserved in evolution and is independent of age, gender, and race.

Treatment dose remains a matter of debate. In a cohort of 181 food‐allergic patients treated with omalizumab, its efficacy was shown to correlate with its dosage per weight but not with its dosage per total IgE and body weight.^12^ For asthma, the omalizumab dosage is determined according to the IgE levels and body weight, and in urticaria a flat rate of 300 mg omalizumab is used but up dosing may be required in patients with higher BMI.^13^ In an ongoing large DBPC study on the use of omalizumab for treatment of food allergy, the dose is based only on body weight (16 mg/kg and 8 mg/kg),^14^ and in the recently completed clinical trials, the same treatment dosage was used as in the asthma treatment protocol.^14^, ^15^, ^16^ The recent approval for omalizumab in IgE‐mediated food allergy uses the same dose recommendations as for asthma,^10^ that is, based on serum total IgE levels and body weight, as this is the approach used in the OUtMATCH study.^9^

## 4 METHODS USED TO DEVELOP THIS POSITION STATEMENT

GA^2^LEN considered the role of omalizumab as part of a comprehensive management plan for people with one or more food allergies. We developed our position based on available evidence and expert opinion.

We drew on a systematic review and meta‐analysis of research on omalizumab alone or used in conjunction with oral immunotherapy.^17^ The review protocol is registered in the International Prospective Register of Systematic Reviews (PROSPERO; number: CRD42021245895). We replicated this analysis using the same search terms to confirm results, since the initial work was industry sponsored. In summary, the reviewers searched seven databases for published and unpublished randomized controlled trials (RCTs), controlled clinical trials and observational studies available as of November 2020. They found 36 relevant studies with a total of 953 children and adults. There were 9 RCTs, 19 controlled clinical trials and 8 observational studies. The purpose of this position paper is not to summarize the systematic review,^17^ but to present some of the key findings as evidence to support our position.

**FIGURE 1:**
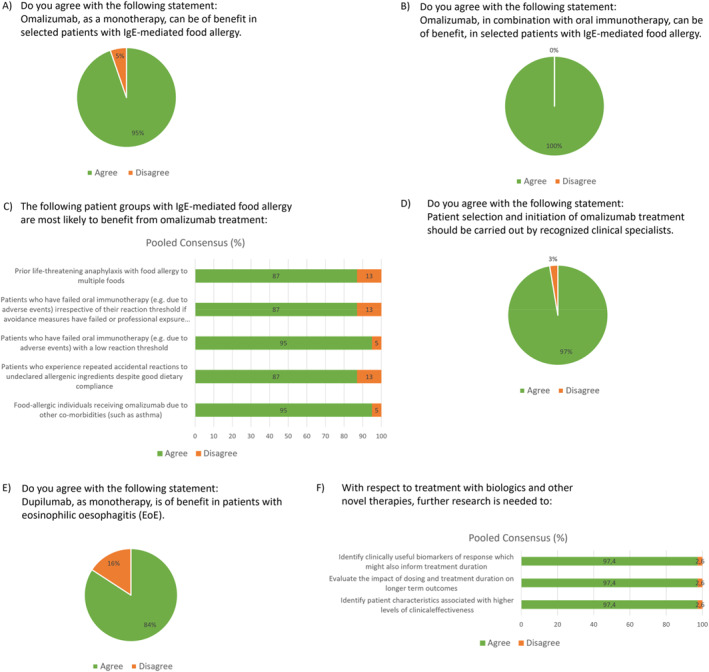
Pooled eDelphi study second round results on the ANACARE expert opinion statements about the use of omalizumab and other biologics for food allergies and eosinophilic esophagitis.

We formed an Expert Group made up of 40 experts from 16 countries. We used an eDelphi process to consider the evidence about the safety and effectiveness of omalizumab in food allergy as well as to discuss the current clinical practice in the different centers. The Expert Group considered the findings from the systematic review,^17^ as well as studies published since which include Stage 1 from the OUtMATCH study,^16^, ^18^ as well as other relevant RCTs, as described in detail below. The main statements of this paper were voted on in the eDelphi, and a 70% consensus was considered as the agreement threshold. The questions were formulated and voted on during ANACARE general assembly meetings in the first half of 2023, and the 4‐point Likert scale for response options (strongly agree, agree, disagree, strongly disagree) was applied. Two rounds of eDelphi were conducted. In the final round, out of the 51 experts who were invited to participate in the questionnaire, 38 responded, giving a response rate of 74.5%. The results of the eDelphi questionnaire were pooled into two groups (agree and disagree) and are presented in Figure 1A–F, all having responses above 70%.

## 5 CURRENT LEVEL OF EVIDENCE FOR USE OF OMALIZUMAB IN FOOD ALLERGY

### 5.1 Omalizumab as monotherapy

The systematic review included 12 studies about using omalizumab as monotherapy to treat people with food allergy.^15^, ^16^, ^19^, ^20^, ^21^, ^22^, ^23^, ^24^, ^25^, ^26^, ^27^, ^28^, ^29^ Overall, omalizumab increased thresholds for allergen reactivity, improved their quality of life, and was not associated with many adverse effects. People allergic to multiple foods or to peanuts were usually at least 2–3 times more able to tolerate these foods compared to the baseline threshold after a course of omalizumab that was administered every 2–4 weeks, for 24 weeks. Studies have reported enhanced tolerance to peanut of 500–6500 mg.^20^, ^21^, ^29^ The findings were more heterogeneous for people allergic to milk or hen's eggs. The phase III OUtMATCH study results indicated that a 16‐week omalizumab treatment was superior to placebo in increasing the reaction threshold for peanut and other common food allergens in individuals with multiple food allergies.^16^ A limitation of the OUtMATCH study is that the primary outcome was the proportion of participants able to tolerate ≥600 mg of peanut protein without experiencing dose‐limiting symptoms at double‐blind food challenge, rather than a change in reaction threshold, which is a more relevant patient outcome. The Expert group also noted the findings from the TOFAC study of 20 children, which reported a significant increase in threshold following 3 months of omalizumab.^15^

The Expert Group considered that while previous studies were small and therefore at greater risk of bias, the OUtMATCH study addressed this limitation. Furthermore, given the consistency of the OUtMATCH results with previous studies, the Expert Group concluded that there is now sufficient evidence to recommend omalizumab as monotherapy for use in selected patients with IgE‐mediated food allergy (Figure 1A). A notable limitation of using omalizumab as monotherapy is that long treatment durations may be required, which are costly and may be inconvenient for patients. Using the safe symptom‐free interval induced by this therapy to induce specific tolerance by oral immunotherapy against the relevant food allergens is therefore likely to be a more cost‐effective use.

### 5.2 Omalizumab in combination with oral immunotherapy (OIT)

Food allergen immunotherapy has recently become available to treat people, especially children, with peanut allergy in many countries worldwide, including the USA and countries in. It has been used in clinical studies for people with other food allergies, mainly milk and egg.^30^

Allergen immunotherapy (AIT) is often associated with adverse effects because people cannot always tolerate the rising doses of the elicit food. Some studies found that 1 in 10 people suffered systemic reactions, especially during the escalation phase.^31^ To address this important issue, recent findings on co‐administration of adjuvants with peanut OIT demonstrated the potential in reducing adverse events, increasing the body's ability to develop sustained unresponsiveness and the long‐term efficacy of AIT.^32^, ^33^, ^34^ This means that immunotherapy must be provided in specialized centers with appropriate medical supervision. The observed adverse effects are one of the barriers why specific immunotherapy has not been fully established for treating food allergies to date.

The systematic review included 22 studies of omalizumab used in combination with oral immunotherapy.^34^, ^35^, ^36^, ^37^, ^38^, ^39^, ^40^, ^41^, ^42^, ^43^, ^44^, ^45^, ^46^, ^47^, ^48^, ^49^, ^50^, ^51^, ^52^, ^53^, ^54^, ^55^ The reviewers concluded that adding omalizumab to the treatment regimen may help people tolerate oral immunotherapy better and have fewer reactions.

The potential advantages of using omalizumab with oral immunotherapy are:

- increased safety and efficacy of oral immunotherapy
- better tolerability of the allergen at higher doses
- reduced duration of the escalation phase of oral immunotherapy
- improvement of asthma, which may be a co‐morbidity and is a recognized co‐factor in severity of food allergy reaction.

Based on the available data, the ANACARE experts are of the opinion that Omalizumab, in combination with oral immunotherapy, can be of benefit in selected patients with IgE‐mediated food allergy (Figure 1B). High quality evidence from large RCTs is currently lacking; however, the Expert Group noted the results from at least 2 large RCTs are expected soon: Stage 2 of OUtMATCH^19^ and the BOOM study, a double‐blind RCT comparing omalizumab to placebo as an adjunct to oral immunotherapy in subjects aged 6–25 years with multiple food allergy.^14^ More robust research is needed to explore the benefit effect size of omalizumab and whether it differs among people with various food allergies. The most effective dosing schedule and duration of treatment also need to be explored further.

### 5.3 Safety profile

The safety and efficacy of omalizumab have been reviewed for other diseases. Godse et al. 2015 reviewed reports of studies involving chronic urticaria patients in which most often no or very mild adverse effects were observed.^56^ The safety in pediatric asthmatic patients was reviewed by Chipps et al. 2017, which resulted in an overall support of the current guidelines that recommend omalizumab as an add‐on treatment in children with uncontrolled persistent allergic asthma.^57^ Omalizumab has been shown to be safe for use in other diseases and may be beneficial to specific food allergic patient subgroups. The use of omalizumab allows flexibility in the treatment of food allergy in which the drug can be tested for a limited period.

## 6 POSITION STATEMENT

We need new approaches to address the heavy burden of food allergies. Based on the Delphi process of expert consensus and a review of the evidence, GA^2^LEN's position regarding omalizumab in IgE‐mediated food allergy is as follows.

### 6.1 Target population

We encourage clinicians to consider whether omalizumab is right for selected patients with IgE‐mediated food allergy. It can cover several food allergies at once and seems equally effective for adults and children. As omalizumab is a non‐specific anti‐IgE, its efficacy is the likely similar for any ethnic group and for any food allergy that is IgE related.

Due to the cost and administration form, we suggest offering omalizumab as an option for those who may benefit most. This includes those at greatest risk of life‐threatening anaphylaxis with food allergy to multiple foods, who have failed oral immunotherapy (e.g., due to adverse events) irrespective of their reaction threshold or low reaction threshold, who experience repeated accidental reactions to undeclared allergenic ingredients despite good dietary compliance, and food allergic individuals receiving omalizumab due to other co‐morbidities (e.g., asthma) (Figure 1C). The use may expand widely once the drug receives regulatory approval for the treatment of food allergy, in which case the direct costs to the patient may decrease considerably.

We advise caution if considering administering omalizumab to pregnant women. However, this should be balanced against the risk of anaphylaxis in pregnancy and thus risk and benefit need to be discussed with the patient.

### 6.2 Treatment regime and evaluation of success

Omalizumab is efficacious in clinical trials but unlikely to be cost‐effective as monotherapy in most patients. Monotherapy may require long‐term treatment (6 months to several years), whereas omalizumab can be used for short‐term treatment (12–18 weeks) to support oral immunotherapy. Specific immunotherapy with food allergens appears effective but is often associated with systemic adverse effects. Omalizumab can reduce this, making immunotherapy more tolerable and safer for a greater number of people. However, for patients with multiple food allergies where immunotherapy may not be practical if there is allergy to a large number of foods, long‐term monotherapy may well be the most reasonable approach.

Currently, the dose of omalizumab for treating food allergy is determined by body weight and total IgE levels.^10^ It should be administered subcutaneously once every 2 or 4 weeks. The fraction of allergen‐specific/total IgE may be useful to predict patients at greater risk of food dosing reactions after reintroduction.^12^ The duration of the treatment cannot be predicted at the time of initiation, but it has been suggested that patients should be reevaluated at regular time intervals of no more than 3 months. Controlled food challenges may be warranted for further decision‐making.

### 6.3 Optimizing safety

Omalizumab has a good safety profile. Admission to the hospital is not required for administration. As this is a biological medicine, it should initially only be administered by a healthcare professional trained to recognize anaphylaxis and in an environment with medications and equipment to respond to a systemic reaction. Anaphylaxis is rare in people who receive omalizumab and is most common after the first few doses. Therefore, people who receive omalizumab should be observed for systemic reactions for 2 h after administration for at least the first three injections. The drug is then licensed for home use if these first doses are well tolerated.

Patients with food allergy who receive omalizumab should still receive the prescription for standard emergency medicine for their underlying food allergy based on the recommendations, such as an adrenaline autoinjector (AAI). They should be trained in how to use the AAI and where to seek help in an emergency.

Based on the available data, the ANACARE experts are of the opinion that patient selection and initiation of omalizumab treatment should be carried out by recognized clinical specialists (Figure 1D).

## 7 OTHER CURRENTLY APPROVED BIOLOGICS FOR FOOD ALLERGY RELATED DISEASES

Dupilumab, a monoclonal antibody that blocks interleukin 4 and interleukin 13, has been approved for eosinophilic esophagitis (EoE), a chronic disease characterized by symptoms of esophageal dysfunction and eosinophil‐predominant inflammation which frequently associated with IgE sensitization to food allergens. However, research has shown that EoE pathogenesis is distinct from IgE‐mediated food allergy and EoE inflammation itself appears to be largely IgE independent. Dupilumab is indicated for the treatment by subcutaneous injection of adult and pediatric EoE patients aged 12 years and older, weighing at least 40 kg, 300 mg weekly. Dupilumab has been evaluated in a three‐part phase 3 double blind placebo controlled clinical trial, which revealed that subcutaneous dupilumab administered weekly improved histologic outcomes and alleviated symptoms of the EoE.^58^ The ANACARE experts are of the opinion that Dupilumab, as a monotherapy, is of benefit to patients with EoE (Figure 1E).

## 8 CONCLUSION

Omalizumab has been used in individual cases of people with food allergy for some time and is now licensed in the United States to treat selected patients with one or more IgE‐mediated food allergies. Currently, the highest level of evidence on the safety and efficacy of omalizumab in treating IgE‐mediated food allergies is available from the OUtMATCH study. Other clinical trials are ongoing but have yet to present their findings or to conclude. As more data become available, we anticipate that omalizumab will be licensed in other countries and more confidently used off‐label. However, there is now sufficient evidence to recommend omalizumab as monotherapy for use in selected patients with IgE‐mediated food allergy, where there is a need to reduce the risk of food‐induced allergic reactions due to patient‐specific factors. This may include patients with repeated unexplained severe anaphylaxis reactions to exposure to food allergens or those in whom a short treatment course might be indicated to reduce risk while receiving omalizumab treatment (e.g., due to travel, occupations with special risk, being in a remote location without medical support). More research needs to be undertaken to assess optimal treatment duration, longer‐term outcomes and cost‐effectiveness prior to being able to recommend a wider indication for use. We await outcomes from ongoing clinical trials to inform future recommendations for omalizumab in combination with oral immunotherapy or as a bridge to real food introduction, which may be more cost‐effective measures to induce longer‐term tolerance.

We encourage considering omalizumab as an option in individual patients with food allergy in collaboration with specialized centers such as the centers of reference of the GA^2^LEN ANACARE network. We encourage considering inducing specific tolerance or at least increasing the threshold of reaction and pre‐treatment with omalizumab can support immunotherapy, but we acknowledge that we do not currently have knowledge about the duration of treatment required and careful supervision on an individual basis is needed. We recommend pursuing further research and future randomized placebo‐controlled trials to identify clinically useful biomarkers of response, which might also inform treatment duration, evaluate the impact of dosing and treatment duration on longer term outcomes, and identify patient characteristics associated with higher levels of clinical effectiveness (Figure 1F).

## AUTHOR CONTRIBUTIONS

**Torsten Zuberbier**: Conceptualization; investigation; funding acquisition; writing ‐ original draft; methodology; validation; writing—review and editing; formal analysis; supervision. **Antonella Muraro**: Conceptualization; methodology; validation; formal analysis. **Ulugbek Nurmatov**: Conceptualization; methodology; validation; formal analysis. **Stefania Arasi**: Conceptualization; methodology; validation; formal analysis. **Katarina Stevanovic**: Conceptualization; writing—original draft; methodology; visualization; writing—review and editing; software; formal analysis; project administration; data curation. **Aikaterini Anagnostou**: Writing—review and editing. **Roberta Bonaguro**: Writing—review and editing. **Sharon Chinthrajah**: Methodology; writing—review and editing. **Gideon Lack**: Writing—review and editing. **Alessandro Fiocchi**: Methodology; writing—review and editing. **Thuy‐My Le**: Writing—review and editing. **Paul Turner**: Methodology; validation; writing—review and editing; formal analysis. **Montserrat Alvaro Lozano**: Writing—review and editing. **Elizabeth Angier**: Writing—review and editing. **Simona Barni**: Writing—review and editing. **Phillippe Bégin**: Writing—review and editing. **Barbara Ballmer‐Weber**: Writing—review and editing. **Victoria Cardona**: Writing—review and editing. **Carsten Bindslev‐Jensen**: Writing—review and editing. **Antonella Cianferoni**: Writing—review and editing. **Nicolette de Jong**: Writing—review and editing. **Debra de Silva**: Writing—original draft; writing—review and editing; methodology; data curation. **Antoine Deschildre**: Writing—review and editing. **Audrey Dunn Galvin**: Writing—review and editing. **Motohiro Ebisawa**: Writing—review and editing. **David M. Fleischer**: Writing—review and editing. **Jennifer Gerdts**: Writing—review and editing. **Mattia Giovannini**: Writing—review and editing. **Josefine Gradman**: Writing—review and editing. **Susanne Halken**: Writing—review and editing. **Syed Hasan Arshad**: Writing—review and editing. **Ekaterina Khaleva**: Writing—review and editing. **Susanne Lau**: Writing—review and editing. **Richard Loh**: Writing—review and editing. **Mika J. Mäkelä**: Writing—review and editing. **Mary Jane Marchisotto**: Writing—review and editing. **Laura Morandini**: Writing—review and editing. **Charlotte G. Mortz**: Writing—review and editing. **Caroline Nilsson**: Writing—review and editing. **Anna Nowak‐Wegrzyn**: Writing—review and editing. **Marcia Podestà**: Writing—review and editing. **Lars K. Poulsen**: Writing—review and editing. **Graham Roberts**: Methodology: Writing—review and editing. **Pablo Rodríguez del Río**: Writing—review and editing. **Hugh A. Sampson**: Writing—review and editing. **Angel Sánchez**: Writing—review and editing. **Sabine Schnadt**: Writing—review and editing. **Peter K. Smith**: Writing—review and editing. **Hania Szajewska**: Writing—review and editing. **Natasa Teovska Mitrevska**: Writing—review and editing. **Alice Toniolo**: Writing—review and editing. **Carina Venter**: Writing—review and editing. **Amena Warner**: Writing—review and editing. **Gary W. K. Wong**: Writing—review and editing. **Robert Wood**: Writing—review and editing. **Margitta Worm**: Writing—review and editing.

## CONFLICT OF INTEREST STATEMENT

Torsten Zuberbier reports honoraria for lectures from Amgen, AstraZeneca, AbbVie, ALK ‐Abelló, Almirall, Astellas, Bayer Health Care, Bencard, Berlin Chemie, FAES Farma, HAL Allergie GmbH, Henkel, Kryolan, Leti, L'Oreal, Meda, Menarini, Merck Sharp and Dohme, Novartis, Nuocor, Pfizer, Sanofi, Stallergenes, Takeda, Teva, UCB, and Uriach; Fees for industry consulting were received from Abivax, Almirall, ,Bluprint, Celldex, Celltrion, Novartis, and Sanofi; in addition he declares non‐paid organizational affiliations: Committee member, “Allergic Rhinitis and its Impact on Asthma” (ARIA), Member of the Board, German Society for Allergy and Clinical Immunology (DGAKI), Head, European Center for Allergy Research Foundation (ECARF), President, Global Allergy and Asthma Excellence Network (GA^2^LEN), and Member, Committee on Allergy Diagnosis and Molecular Allergology, World Allergy Organisation (WAO). Antonella Muraro reports speaker's fees from Viatris, DVB Technologies, Aimmune, Novartis, and Nestle Health Sciences, and is a non‐paid committee member of “GA2LEN Executive Committee” and “GA2LEN ANACare Steering Committee.” Ulugbek Nurmatov declares no conflict of interest. Stefania Arasi reports contracts from Bambino Gesu’ Children Research Hospital, speaker's fees from Ulrich and DBV, participation on the advisory board for Novartis and AIMMUNE, and is a non‐paid committee member of EAACI. Katarina Stevanovic declares no conflicts of interest. Aikaterini Anagnostou reports consulting fees from Novartis, Genentech, ALK; speaker's fees from EPG Health, MJH, Adelphi, Aimmune Therapeutics, Genentech, FARE, Medscape, Innovation horizons; travel fee support from Novartis, Medscape, multiple allergy societies, and participation on advisory boards for Ready set food, Novartis, Genentech, and Bryn. Roberta Bonaguro declares no conflicts of interest. Sharon Chinthrajah reports grants from Consortium for Food Allergy Research (CoFAR), National Institute of Allergy and Infectious Disease (NIAID), Food Allergy Research and Education (FARE); is an advisory board member for Alladapt Immunotherapeutics, Novartis, Allergenis, Intrommune Therapeutics, Phylaxis, Genentech, and Blueprint Therapeutics; is a stockholder of Intrommune Therapeutics. Gideon Lack reports grants from the National Institute of Allergy and Infectious Diseases (NIAID, NIH) and National Peanut Boards (NPB); consulting fees from Novartis, DBV Technologies, Reckitt Mead Johnson, and ALK Abello; speaker's fees from DBV Technologies, Aimmune, and EPG Health; and is a shareholder of DBV Technologies and Mighty MissionMe. Alessandro Fiocchi reports grants from Novartis, Ferrero, Sanofi, Stallergenes, Danone, and Aimmune; consulting fees from Abbott and Ferrero; speaker's fees from Sanofi; and non‐paid committee member of World Allergy Organization (WAO) and American Academy of Allergy Asthma and Immunology (AAAAI). Thuy‐My Le reports speaker's fees from Thermofisher and Abbvie. Paul Turner reports grants from the Medical Research Council, Food Standards Agency, JM Charitable Foundation, National Institute for Health and Care Research (NIHR)–Imperial Biomedical Research Center, outside the submitted work, and personal fees from the UK Food Standards Agency, DBV Technologies, Aimmune Therapeutics, Allergenis, and ILSI Europe outside the submitted work. Montserrat Alvaro Lozano declares no conflict of interest. Elizabeth Angier reports support for travel expenses and honoraria from Gp Lecture; is a non‐paid member of Primary Care group members BSACI, Anapyhylaxis UK, and Allergy UK. Simona Barni reports speaker's fees from Nutricia and Sanofi. Phillippe Bégin reports payments to institutions for clinical trials from Novartis, DBV Technologies, and Sanofi; consulting fees from ALK, Novartis, DBV Technologies, and Pfizer; and speaker's fees from ALK and Novartis. Barbara Balmer‐Webber reports consulting fees from ALK, Novartis, Sanofi, and Allergopharma; speaker's fees from Thermo Fisher, Novartis, Sanofi, and Menarini. Victoria Cardona declares no conflicts of interest. Carsten Bindslev‐Jensen reports grant from Novartis and consulting fees from ALK Abello and Novartis. Antonella Cianferoni declares no conflicts of interest. Nicolette de Jong declares no conflicts of interest. Debra de Silva declares no conflicts of interest. Antoine Deschildre reports consulting fees from Novartis, ALK, GSK, Sanofi, Regeneron, Aimmune Therapeutics, Nestlé Health Science, Stallergènes‐Greer, Viatris, Celltryon; speaker's fees from Novartis, ALK, GSK, Sanofi, Aimmune Therapeutics, DBV Technologies, Nestlé Health Science, Viatris; travel costs support from ALK, Sanofi, Stallergenes Greer, Novartis, Astra‐Zeneca, Aimmune Therapeutics, Celltryon; participation on a data safety monitoring board for the BOOM study. Audry Dunn Galvin reports consulting fees from Novartis; speaker's fees from Novartis and DBV, support for travel costs from Novartis and DVB, and is a non‐paid member of GA2LEN and Anaphylaxis Ireland. Motohiro Ebisawa reports consulting fees from Novartis, ARS‐Pharmaceuticals, and Sanofi; speaker's fees from Viatris. David M. Fleischer reports grants to institutions from ARS‐Pharmaceuticals and DBV Technologies; Royalties from UpToDate; consulting fees from Genentech, ARS‐Pharmaceuticals, Bryn Pharma, DBV Technologies, and Nasus Pharma; speaker's fees from Genentech; and is a nonpaid member of AAAAI Division Director's Committee, National Peanut Board Medical Advisory Council, Food Allergy and Anaphylaxis Connection Team Medical Advisory Board; and stock owner of Grow Happy. Jennifer Gerdts reports on her honorarium as a member of the Novartis Global Patient Advisory Group. Mattia Giovannini reports speaker's fees from Sanofi. Josephine Gradman declares no conflicts of interest. Sussanne Halken reports speaker's fees from ALK Nordic, Nestlé Purino, and Abigo and personal honoraria as a member of an independent data monitoring committee for Stallergenes. Syed Hasan Arshad reports a grant from the Natasha Allergy Research Foundation. Ekaterina Khaleva declares no conflicts of interest. Susanne Lau reports grant to institution for clinical trial from DBV; speaker's fees from Sanofi‐Aventis and DBV, travel costs support from DBV and Allergopharma, and advisory board honoraria from Sanofi Aventis, DBV, and Leo Pharma. Richard Loh declares no conflicts of interest. Mika J. Mäkelä declares no conflicts of interest. Mary Jane Marchisotto declares no conflicts of interest. Laura Morandini declares no conflicts of interest. Charlotte G. Mortz reports research grants from Novartis. Caroline Nilsson reports grants to institutions from Aimmune Therapeutics and grants for material in a study from Thermo Fisher; speaker's fees from ALK, Thermo Fisher, and GSK; and is a member of the advisory board for Aimmune Therapeutics. Anna Nowak‐Wegrzyn reports speaker's fees from Genentech. Marcia Podesta reports grants from DBV Technologies, Viatris, and Novartis and is a non‐paid member of the Food Allergy Italia, GA2LEN ANAcare Patient's Advocates Team, EACCI Patient Organisation Committee, EACCI Ethics Committee, FAO WHO Codex Alimentarius Commission, and EAACI food allergy‐related task forces and guideline groups. Lars K. Poulson declares no conflict of interest. Graham Roberts reports grants from the National Institutes of Health Research, National Institute of Health, and Action Medical Research; consulting fees from ALK‐Abello, Thermo Fisher, and Astra Zeneca; and is the president of the British Society of Allergy and Clinical Immunology. Pablo Rodrguez del Ro reports a grant from Aimmune Therapeutics, DBV, FAES, and Novartis; speaker's fees from GSK, FAES, Novartis, ALK‐Abelló, LETI, Sanofi, Stallergenes, DBV, EPG‐Health, and Roxall. Hugh A. Sampson report grants for research to institution from National Institutes of Health (NIAID), Food Allergy Research and Education (FARE), and Allergenis; textbook royalties from Wiley/Blackwell and Elsevier; consulting fees from DBV Technologies, N‐Fold LLC, Alpina Biotechnology, Siolta Therapeutics, and AbbVie; honoraria from AAAAI and ACAAI; travel costs support from AAAAI and DBV Technologies, institutional patents for Peptides And Methods For Detecting Egg Allergies, Peptides And Methods For Detecting Peanut Allergies, Methods for characterizing antibody binding affinity and epitope diversity in food allergy, Compounds, extracts and methods from Chinese medicinal herb Sophora flavescens that inhibit airway contractions; stockholder of DBV Technologies and N‐Fold LLC. Angel Sanchez reports consulting fees from Aimmune Therapeutics; fees for participation on the Global Food Allergy Council 2022/2023 by Novartis. Sabine Schnadt declares no conflicts of interest. Peter K. Smith reports grants paid to institutions from Sanofi and GSK; honoraria from Viatris and Novartis; and is a non‐paid member of the AusEE patient group advisory board. Hania Szajewska reports honoraria from Arla, BioGaia, Biocodex, Danone, Dicofarm, Nestlé, NNI, Nutricia, Mead Johnson, and Novalac. Natasa Teovska Mitrevska reports declare no conflict of interest. Alice Toniolo declares no conflicts of interest. Carina Venter reports honoraria from Dannone, Reckitt, Neste Nutrition Institute, and Abbott; research grant to institution from Reckitt; and is a stockholder of Growhappy. Amena Warner reports employment by the patient charity Allergy UK. Gary W. K. Wong reports that he is a non‐paid president of the Hong Kong Institute of Allergy. Robert Wood reports grants from Aimmune, ALK, DBV, Genentech, Novartis, NIH, Siolta, and Aravax and consulting fees from Genentech. Margitta Worm reports consulting fees from Novartis Pharma GmbH, Sanofi‐Aventis Deutschland GmbH, DBV Technologies S.A, Aimmune Therapeutics UK Limited, Leo Pharma GmbH, AstraZenceca GmbH, ALK‐Abelló Arzneimittel GmbH, Lilly Deutschland GmbH, Kymab Limited, Amgen GmbH, Abbvie Deutschland GmbH and Co. KG, Pfizer Pharma GmbH, Mylan Germany GmbH (A Viatris Company), Boehringer Ingelheim Pharma GmbH and Co. KG, GlaxoSmithKline GmbH and Co. KG, Almirall S. A., Amgen GmbH, Pfizer Deutschland GmbH, Bristol‐Myers Squibb GmbH and Co. KGaA and FomF GmbH; honoraria from Novartis Pharma GmbH, Sanofi‐Aventis Deutschland GmbH, DBV Technologies S.A, Aimmune Therapeutics UK Limited, Leo Pharma GmbH, AstraZenceca GmbH, ALK‐Abelló Arzneimittel GmbH, Lilly Deutschland GmbH, Kymab Limited, Amgen GmbH, Abbvie Deutschland GmbH and Co. KG, Pfizer Pharma GmbH, Mylan Germany GmbH (A Viatris Company), Boehringer Ingelheim Pharma GmbH and Co. KG, GlaxoSmithKline GmbH and Co. KG, Almirall S. A., Amgen GmbH, Pfizer Deutschland GmbH, Bristol‐Myers Squibb GmbH and Co. KGaA and FomF GmbH.

## Data Availability

Data sharing is not applicable to this article as no new data were created or analyzed in this study.
